# Artificial Shelters as a Monitoring and Conservation Tool for Terrestrial Breeding Frogs

**DOI:** 10.1002/ece3.73215

**Published:** 2026-03-12

**Authors:** Jordy Groffen, Lyanne Brouwer, Myles H. M. Menz, Conrad J. Hoskin

**Affiliations:** ^1^ College of Science and Engineering James Cook University Townsville Queensland Australia

**Keywords:** microclimate, microhabitats, nursery frogs, shelter design, shelter occupancy, thermal buffer

## Abstract

Amphibians are among the most threatened vertebrate groups, yet monitoring cryptic, fossorial species is difficult and often risks disturbing both their microhabitats and the individuals themselves. We tested whether artificial shelters could serve as a monitoring and potential conservation tool for cryptic fossorial amphibians in natural habitats. We deployed two different artificial shelter designs (concrete and wood) in the Australian Wet Tropics and assessed the number of *Austrochaperina robusta* , a terrestrial‐breeding microhylid frog, using them over 2 years. Monthly shelter surveys recorded the highest frog numbers in the cooler, drier months, indicating clear seasonal trends. Sliding‐window analyses showed that frog numbers under concrete, but not wooden, shelters declined with warmer minimum temperatures over the 6 days pre‐survey. Frogs used concrete shelters more (75% of records) compared to wooden shelters. This was partly because the concrete shelters did not degrade with time, but also likely reflects thermal buffering benefits. Microclimate logging indicated that concrete shelters provided greater daytime heat storage and slower nocturnal heat release than the wooden shelters. Invertebrate presence was weakly negatively associated with frog numbers, while greater understory cover and closer shelter spacing were stronger positive predictors of frog abundance. While most observations were of adult frogs, juveniles and metamorphs were also observed under both shelter designs. Additionally, six egg clutches were recorded, all under concrete shelters. Collectively, these results show that artificial shelters, especially concrete designs, offer a robust, low‐impact method for long‐term monitoring of fossorial frogs and may contribute to conservation by providing thermally favourable refugia and protection from disturbance.

## Graphical abstract

Artificial concrete and wooden shelters were tested as low‐impact tools for monitoring a cryptic terrestrial‐breeding frog, *Austrochaperina robusta* , in the Australian Wet Tropics. Frogs overwhelmingly preferred concrete shelters, which provided stronger thermal buffering, supported higher occupancy across seasons, and were the only shelters used for egg‐laying. These results show that well‐designed artificial shelters can effectively monitor fossorial frogs while also offering conservation benefits through improved refuge and breeding microhabitats.

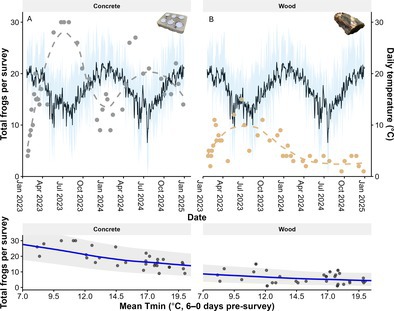

## 1 Introduction

A key challenge in monitoring rare herpetofauna (amphibians and reptiles) is balancing the positives of survey activities against potential disturbance and damage to organisms and their habitats (Bodinof Jachowski et al. 2020). This is particularly the case for the many reptiles and amphibians that have cryptic life histories and shelter under or in logs, rocks, soil, leaf litter or plants (Stewart 1995). Many of these species are rarely surface‐active, making them hard to study and often requiring invasive searches through habitats (e.g., lifting logs). This can damage the microhabitats and cause disturbance and potential injury or death to individuals (Hesed 2012). Additionally, active searches take time and expertise (Glorioso et al. 2022) and can suffer from observer biases (Dodd 2016). Artificial shelters—human‐made structures designed to provide secure spaces for species to seek refuge and/or breed (Bodinof Jachowski et al. 2020; Michael et al. 2012; O'Brien et al. 2023, 2025)—offer a non‐invasive alternative by allowing repeated monitoring with minimal disturbance, provided they replicate key ecological features required by the target species.

Microhabitats are essential for refuge during unfavourable environmental conditions (e.g., dry, cold or hot periods) and for supporting vital life history stages such as reproduction and parental care. Optimal microhabitats may become increasingly important under climate change, as they can buffer animals from extreme temperatures or droughts (Scheffers et al. 2014). This is particularly true for direct‐developing terrestrial breeding amphibians, which are predicted to be more vulnerable to rising temperatures than aquatic breeding amphibians, as free‐swimming tadpoles are able to seek deeper, cooler temperatures, unlike direct‐developing larvae, which are confined within the egg (Scheffers et al. 2013).

These refuges could be natural (e.g., adding logs or rocks) or artificial. Examples of artificial shelters used in herpetological studies include cover boards, arboreal shelters, PVC pipes, aquatic concrete shelters, perforated bricks, and leaf‐litter analogues (e.g., Boughton et al. 2000; Button, Bodinof Jachowski, et al. 2020; Button, Hallagan, et al. 2020; Grant et al. 1992; Hopkins et al. 2023; Michael et al. 2004, 2012; Nordberg and Schwarzkopf 2015; Waddle et al. 2024; Waldron et al. 2003). These approaches have generally been successful within their respective systems, supporting detection, monitoring, and in some cases reproduction or long‐term conservation outcomes. Despite widespread use in other taxa (e.g., avian studies; e.g., Hope et al. 2023; Thompson et al. 2023; Marcus et al. 2024), their use in reptile and amphibian studies has largely been applied in taxon‐ or system‐specific contexts, highlighting an opportunity to expand non‐invasive monitoring tools within herpetology.

Artificial shelters may be able to provide better‐buffered refuge and breeding sites than natural shelters as their size, material, and design can be adjusted to provide cooler, warmer or moister microhabitats (reviewed Cowan et al. 2021; Watchorn et al. 2022). This could help species survive in increasingly warm, cold or dry environments with more frequent and extreme climatic weather events (e.g., Cowan et al. 2021; Watchorn et al. 2022). Additionally, artificial shelters can be designed to enable low‐impact and less labour‐intensive monitoring of amphibian populations compared to active searches involving the turning of natural cover (e.g., logs and rocks) or pitfall trapping (Sutherland et al. 2016; Hesed 2012; Bodinof Jachowski et al. 2020; Valdez et al. 2021). Reducing potential impacts on individuals and habitats is particularly important for threatened species, especially during breeding periods.

Despite their potential benefits, artificial shelters have not been thoroughly tested for amphibians (Shoo et al. 2011). Further testing of their design and effectiveness will be valuable for developing on‐ground conservation strategies both in the wild and in captive breeding programs. All artificial shelter studies on amphibians in Australia have been performed in heavily modified landscapes, primarily in south‐eastern Australia, where natural refuge sites have been degraded or lost (Hecht‐Kardasz et al. 2012; Kay et al. 2017; Michael et al. 2004, 2012, 2019). To our knowledge, no studies have investigated the use of artificial shelters in natural (unmodified) habitats. This is surprising given that the vast majority of Australia's threatened frog species occur in the relatively undisturbed mountain regions of eastern and north‐eastern Australia (Geyle et al. 2021), with climate change being one of the biggest threats, and understanding the use of microhabitats by these species has been identified as a conservation research priority (Gillespie et al. 2020). Of particular relevance are the microhylid frog species (*Cophixalus* and *Austrochaperina*) found in the Wet Tropics World Heritage Area of north‐eastern Australia. This diverse group of small, cryptic, terrestrial‐breeding montane species is highly threatened by climate change, with five species listed as Critically Endangered and one as Endangered under the Australian Environmental Protection and Biodiversity Conservation Act (1999), all due to climate‐related impacts.

Here, we examine whether artificial shelters can serve as effective tools for monitoring and conserving cryptic, fossorial species such as Australian microhylid frogs, with potential broader applications to amphibians globally. Specifically, we aim to: (1) compare year‐round patterns of frog abundance and relative shelter use between concrete and wooden shelters, (2) assess whether the shelters were used as oviposition sites, and (3) examine habitat characteristics and environmental factors (e.g., canopy cover, temperature buffering, and precipitation) to assess their influence on shelter use. Based on the fossorial ecology and terrestrial breeding mode of microhylid frogs, we expect frog numbers to be higher in thermally buffered shelters, particularly during cooler periods, and for oviposition to occur primarily in shelter types providing more stable microclimatic conditions. More generally, previous studies have shown that habitat quality and structure (e.g., Bell and Bell 1994; Chambers 2008; Arntzen et al. 2017), as well as environmental conditions (Jung et al. 2024; Leung et al. 2024; Reniers et al. 2015), play a key role in influencing amphibian numbers, particularly for surface‐dwelling species with limited dispersal ability. Our study focuses on the robust whistling frog ( *Austrochaperina robusta* ), a common species in the upland rainforests of the southern Wet Tropics, with a small home range (Groffen et al. 2025). This species serves as a representative model for several threatened microhylid species in north‐east Australia, because it shares key ecological and biological traits, such as breeding strategies (direct‐developing terrestrial breeders) and habitat (mid to high elevation mountain ranges in the Wet Tropics; Hoskin 2004), with these more vulnerable species, making it suitable for developing broader monitoring and conservation strategies.

## 2 Material and Methods

### 2.1 Study Species and Area

Australian microhylid frogs are small‐bodied (≈2 cm), fossorial or scansorial species with a cryptic lifestyle, typically under or in leaf litter, soil, logs, and rocks. They are terrestrial breeders with direct development (i.e., tadpoles develop within the egg capsules). These cryptic species are difficult to study and monitor, particularly outside their short calling periods, resulting in limited information on their life history and breeding ecology (Anstis et al. 2011; Hoskin 2004).

Data was collected in the Australian Wet Tropics, a topographically complex series of mountains, with isolated cool upland habitats surrounded by warm lowlands (Shoo et al. 2011; Shoo and Williams 2004). The study was conducted in the upland rainforest (850–950 m) of the Paluma Range (−19.010886°, 146.206725°), an area that supports high densities of two microhylid frog species, *A. robusta* and *Cophixalus australis* (Hoskin 2004), and is accessible year‐round, allowing consistent sampling across seasons.

### 2.2 Artificial Shelters

**FIGURE 1:**
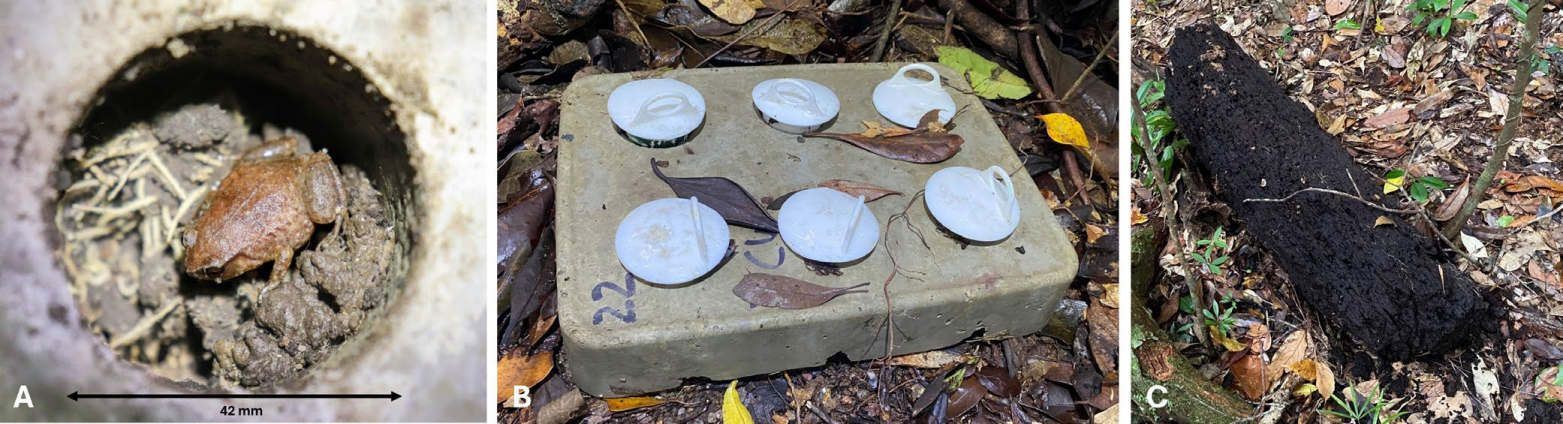
(A) An adult *Austrochaperina robusta* in a concrete shelter, (B) an example of a concrete shelter, and (C) a wooden shelter. Note the plugs placed in the concrete shelter (B) to allow for observation when removed (A).

Two artificial shelter types were constructed: one from wood and one from concrete. The wooden shelters were made by splitting logs found at the site (e.g., from a tree fall near a road or path; Figure 1) and mimicking a natural refuge. By splitting the log in half, we produced a flat surface to place against the ground. The wooden shelters varied depending on available fallen timber (equal size pieces of wood could not be brought to the site due to restrictions on bringing wood into the national park). Shelter size was standardised as much as possible, with wooden shelters averaging 48.8 ± 14.3 SD cm in length, 19.7 ± 7.1 cm in width and 14.6 ± 6.8 cm in height (mean ± SD; Groffen and Hoskin 2024). The concrete artificial shelters were constructed at James Cook University (Townsville, Queensland, Australia). Concrete was chosen due to durability, ease of standardisation, and potential to buffer temperature. The concrete blocks mimic rocks, which occur at the site and under which *A. robusta* is known to shelter. The shelters consisted of rectangular concrete blocks (29.5 × 22.5 × 4.5 cm), with six rounded (42 mm diameter) chambers that each had two narrow entrances/exits (Groffen and Hoskin 2024). Each chamber was accessible via a removable plug, allowing inspection for frog presence and nesting, and camera deployment (Groffen and Hoskin 2024). Detailed construction methods are provided in Groffen and Hoskin (2024).

Thirty wooden and 30 concrete shelters were placed within an approximately 124 m^2^ area in the Paluma range, in an area known for high densities of *A. robusta* and *Cophixalus australis* . Shelters were installed between November 2022 and January 2023. Leaf litter was cleared, and each shelter was placed directly on the soil to ensure it lay as flat as possible. The shelters were always placed in pairs, with a concrete shelter and a wooden shelter positioned within 110 cm of each other, to facilitate direct comparison of shelter use (Figure 1). There was at least 25 m between shelter pairs.

### 2.3 Shelter Checks

We conducted shelter surveys every 4 weeks during the dry and presumed non‐breeding season (March–November), and every 3–7 days during the wet and presumed peak breeding season (December–February) between February 2023 and January 2025. To determine if shelters were occupied, we gently tilted them on their side and inspected the ground and tunnels for frogs and frog nests, while simultaneously recording shelter occupancy by non‐target species (e.g., invertebrates). If tunnels or chambers were blocked by worm castings (small piles of soil), we unblocked them so they remained accessible to the frogs.

### 2.4 Weather Data

Weather data for Paluma was obtained between 1 January 2023 and 31 December 2024, from two sources. Hourly temperature and humidity data were obtained from a private landowner's weather station (weather station ID: IPALUM7, Weather Underground, −19.0167°, 146.167°), located approximately 10 km from the study site. Daily rainfall data were obtained from the Australian Bureau of Meteorology weather station at Paluma (Bureau of Meteorology 2025, weather station 032064), located adjacent to the study site.

### 2.5 Microclimates

At seven shelter pairs, we installed identical logger (iButtons) arrays to quantify thermal buffering, defined as the difference between ambient air temperature and the temperature within each microhabitat (i.e., ambient minus microhabitat temperature). This allowed comparison of buffering among four microhabitats within the same 2 × 2 m plot: beneath the concrete shelter, beneath the wooden shelter, under leaf litter, and in surface soil (microhabitats known to be used by this species; Groffen et al. 2025). From 1 January to 31 December 2024, soil loggers were buried at 4 cm in stainless‐steel mesh tea strainers, and leaf‐litter loggers were placed in mesh strainers under the leaf litter. To benchmark microhabitat buffering against air conditions, ambient temperature was recorded 1.2 m above ground with an iButton suspended in a tea strainer and rain‐shielded by a plastic cup. For analysis, temperature records were pooled by microhabitat type across the seven sites (Figure 4).

### 2.6 Habitat Characteristics

All shelters were under ‘closed canopy’ in the upland rainforest, and habitat measurements were taken to characterise the surrounding microhabitat at each shelter. Measurements were taken within 48 h after installing the shelters, using a 2 × 2 m plot centred around each concrete shelter. Subsequent tracking confirmed that *A. robusta* has a very small home range (average 0.46 ± 0.20 m^2^; Groffen et al. 2025), validating that this plot size was appropriate. In each plot, 11 habitat characteristics were measured inside the 2 × 2 m plot (listed in Table 1). Canopy cover for each plot was measured using the Canopeo app (Patrignani and Ochsner 2015), using an iPhone 11 held approximately 1.2 m above ground level above the concrete shelter. Ground cover was estimated as the percentage area of bare ground, coarse woody debris, trees, and leaf litter within each 2 × 2 m plot. Soil texture was determined using the ribbon technique (categories between sand and heavy clay; NSW Department of Primary Industries 2014), with one measurement per plot. The distance between shelters and the concrete shelter and closest object were measured using a measuring tape. Invertebrates beneath shelters were classified by body type and behaviour, based on literature and our observations, into potential predators, hard‐bodied, soft‐bodied, and social insects (Table 2), without species‐level identification.

**TABLE 1:** Climatic and habitat structure variables were measured to assess frog numbers in artificial shelters. Climate variables (temperature, humidity, and rain) were recorded with weather stations, while habitat structure was quantified in 2 × 2 m plots surrounding each shelter set using estimates, canopy photography, or measurement tape.

Category	Variable description	Measurement tool	Measurement location
Climate	Minimum daily ambient temperature (°C) (*T*_min_)	Weather station IPALUM7, Weather Underground	−19.018166°, 146.109179°. Mt Zero
	Maximum daily ambient temperature (°C) (*T*_max_)	Weather station IPALUM7, Weather Underground	−19.018166°, 146.109179°. Mt Zero
	Average daily humidity (%)	Weather station IPALUM7, Weather Underground	−19.018166°, 146.109179°. Mt Zero
	Daily rainfall (mm)	bom.gov.au Station 032064 Paluma	−19.007544°, 146.205649°, Paluma town
Habitat structure	Canopy cover (%)	Canopeo app (Patrignani and Ochsner 2015)	1.2 m above every concrete shelter
	Floor cover of bare ground (%)	Estimates	2 × 2 m plot around each shelter set
	Floor cover of coarse woody debris and logs (%)	Estimates	2 × 2 m plot around each shelter set
	Floor cover of leaf litter (%)	Estimates	2 × 2 m plot around each shelter set
	Floor cover of rock (%)	Estimates	2 × 2 m plot around each shelter set
	Floor cover of understory plants (%)	Estimates	2 × 2 m plot around each shelter set
	Number of small trees (< 25 cm circumference)	Measure the tape circumference, then count	2 × 2 m plot around each shelter set
	Number of large trees (> 25 cm circumference)	Measure the tape circumference, then count	2 × 2 m plot around each shelter set
	Distance between the two shelter types (cm)	Measurement tape	Between shelters in each shelter set
	Distance between shelters and the closest object	Measurement tape	Between every shelter and the closest object
Soil properties	Soil type	Ribboning technique (NSW Department of Primary Industries 2014)	Once in the 2 × 2 m plot around the shelter set

**TABLE 2:** Grouping of invertebrates and total number of records (absence/presence) from monthly shelter checks conducted between February 2023 and January 2025. Invertebrates beneath shelters were classified by body type and behaviour, based on literature and our observations, into potential predators, hard‐bodied, soft‐bodied, and social insects (Table 2), without species‐level IDs.

Group name	Species included	Shelter type	
		Concrete	Wooden
Potential predators	Funnel web spiders, Huntsman spiders, Centipedes, Scorpions	264	156
Hard‐bodied	Beetles, Cockroaches, Crickets, Earwigs, Millipedes	219	291
Soft‐bodied	Earthworms, Snails, Semi‐slugs, Velvet worms	81	96
Social insects	Ant nests, termites	135	84

### 2.7 Statistical Analyses

All data analyses were conducted in R version 4.1.0 (R Core Team 2024) and figures were created using ggplot2 (Wickham 2016). We first aimed to predict seasonal patterns in frog numbers under the shelters to inform monitoring strategies and conservation planning. To predict frog numbers across months, we used generalised linear models (GLMs) with a Poisson error distribution. Monthly averages were calculated across both survey years (2023–2024) for each shelter type and life stage, providing a single mean count per month. We modelled the average number of adult, juvenile, and metamorph frogs, as well as the average total number of frogs per shelter type (concrete or wood) per month, as response variables, with the predictor month included as a fixed factor. Each life stage and shelter type was analysed separately to capture potential differences in seasonal use patterns. Predictions of monthly frog numbers per life stage and shelter type were generated using the predict() function with type = ‘response’ to obtain values on the original count scale.

Next, we aimed to identify the climatic, environmental, and habitat factors that influence frog numbers under the shelters. We first determined how weather affects frog numbers, through performing sliding‐window analyses (Bailey and Van de Pol 2016; van de Pol et al. 2016). This allowed us to identify which weather variables (minimum [*T*_min_] and maximum [*T*_max_] temperature, humidity, and rainfall) over which time‐period best explained variation in shelter use of concrete and wooden shelters. To identify the critical weather variable, we used the R package ‘climwin’ (Bailey and Van de Pol 2016) and systematically explored all possible combinations of time windows for the previous 30 days (the time interval between surveys). The climwin function iteratively identifies which time window (start and end day) yields the best model fit based on Akaike's information criterion (AICc; Akaike 1973; Burnham and Anderson 2002), with lower AICc values indicating stronger support from the data. Given the large number of time windows being considered, we used randomisation techniques to exclude false‐positive results (van de Pol et al. 2016). To test for collinearity and confirm the presence of multiple signals, the best‐supported window of one weather variable was added to the baseline model of another, and vice versa, after which the windows were refitted (van de Pol et al. 2016). The baseline model included the number of frogs as the response variable, fitted using a generalised linear model with a Poisson distribution and log link. The climate window analysis was conducted independently for concrete and wooden shelters, as frog number patterns and responses to weather conditions were expected to differ between shelter types.

To assess whether microclimate differences between the two shelter types influenced the number of frogs under the shelters, we performed similar sliding‐window analyses using microclimate data acquired through the temperature loggers (iButtons).

Following the identification of the best supported weather window, we used a model selection approach to identify the relative importance of weather, environmental, habitat, and shelter characteristics for shelter usage (i.e., frog numbers) (see for details Table 1). To do so, the total number of frogs observed per shelter per survey was fitted as a response variable in a generalised linear mixed model (GLMM) with a Poisson distribution and a log‐link function (Bates et al. 2015). The critical weather variable derived from our sliding window analyses, and environmental, habitat, date, and shelter characteristics were included as covariates. Due to the difficulty of obtaining accurate counts for some invertebrate taxa (such as ant nests), invertebrate data were recorded as present or absent. To account for repeated measures within shelters across time, shelter ID was included as a random intercept. All continuous predictors were standardised to z‐scores to facilitate model convergence and improve interpretability. Model fit and convergence were assessed using the ‘performance’ package (Lüdecke et al. 2021) and variance inflation factors (VIF) were calculated to assess collinearity. Soil type was removed from the final model, as almost half of the shelters were on sandy loam soil, which resulted in high multicollinearity (VIF = 11.2). Model selection was performed using an all‐subset approach with the ‘dredge’ function in the ‘MuMIn’ package (Barton 2019). Models were fitted using maximum likelihood estimation. The proportion of variance explained by the model was assessed using marginal and conditional R^2^ values (Nakagawa and Schielzeth 2013), which represent the variance explained by fixed effects and by both fixed and random effects combined, respectively.

## 3 Results

Forty‐seven shelter surveys were completed between January 2023 and January 2025. During these surveys, we recorded a total of 817 observations of microhylid frogs under the shelters. *Austrochaperina robusta* accounted for 809 of these observations (99%), with only eight observations of *Cophixalus australis* .

### 3.1 Year‐Round Shelter Use Patterns

**FIGURE 2:**
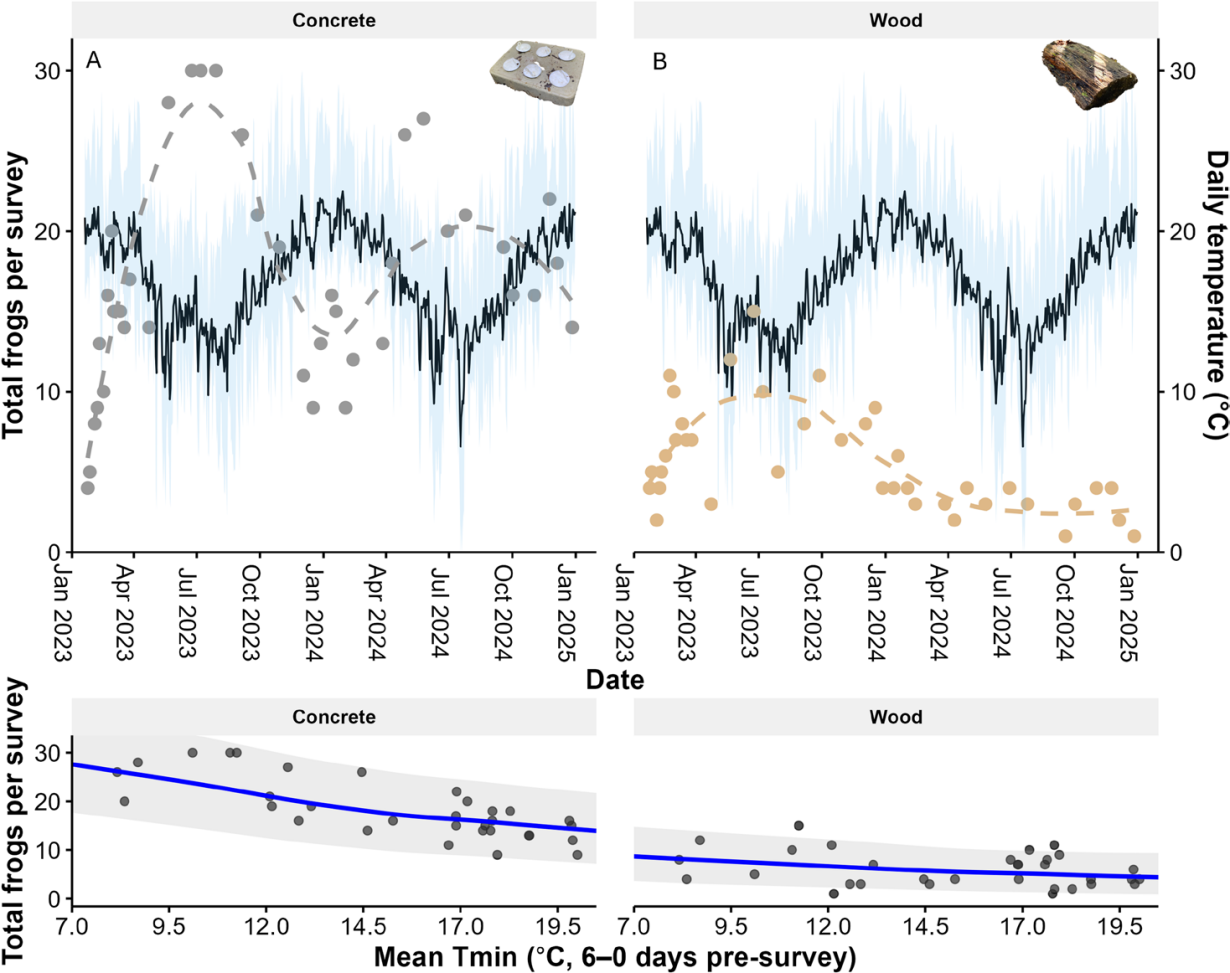
Seasonal patterns of frog numbers under the shelters. The top panels show total number of *Austrochaperina robusta* counts per survey, separated by shelter type (A: Concrete = grey dots, B: Wood = brown dots), with dashed LOESS trendlines indicating frog number changes over time. The black line represents the average daily temperature, while the blue ribbon illustrates the daily minimum (*T*_min_) and maximum (*T*_max_) temperatures. The bottom panels show the observed and model‐predicted (blue line) relationships between mean *T*_min_ (6 days pre‐survey) and total frogs per survey for each shelter type, based on model 1 in Table 3. Grey shaded areas denote 95% prediction intervals.

**FIGURE 3:**
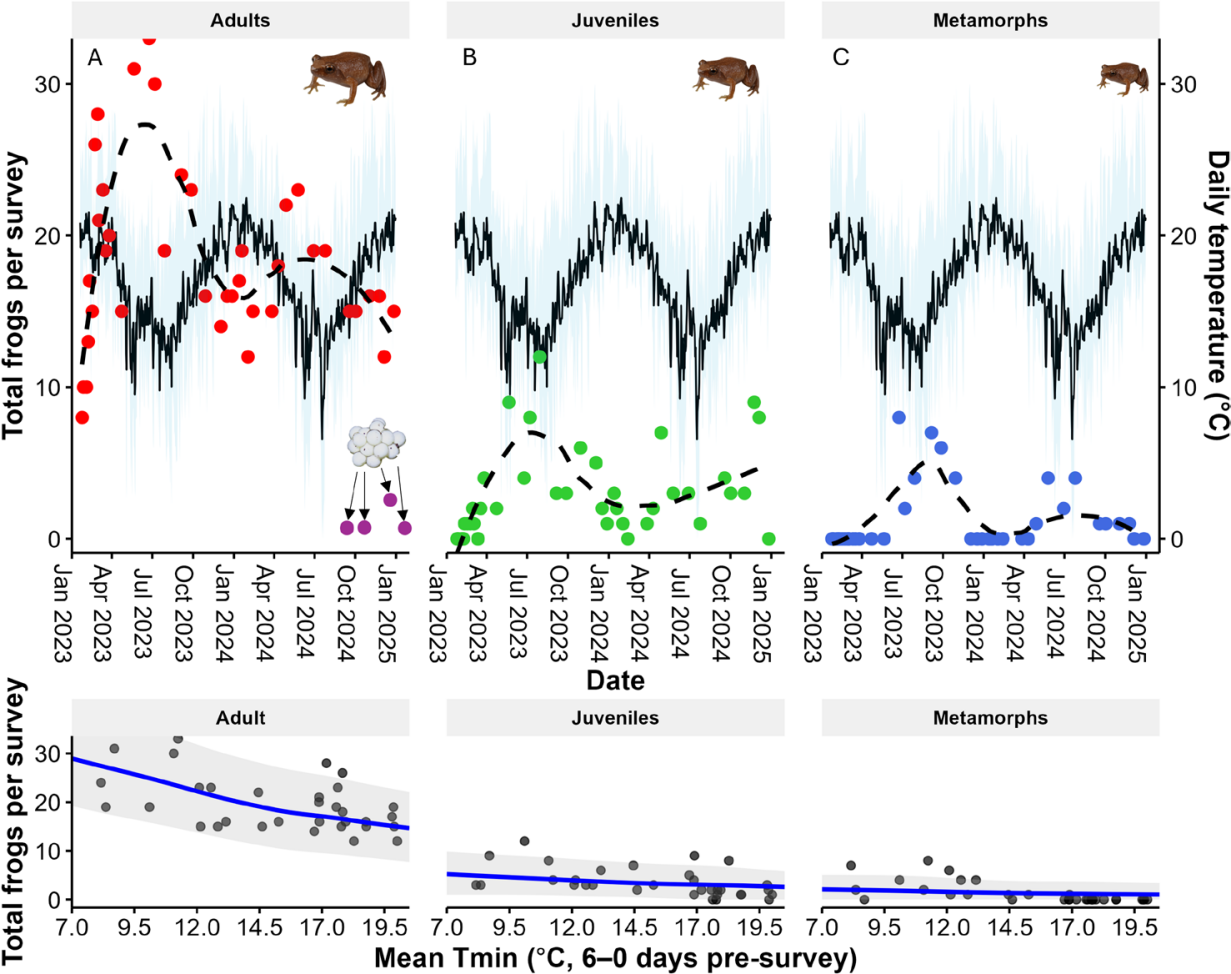
Seasonal patterns by life stage. The top panels show the total number of *Austrochaperina robusta* individuals recorded per survey, separated by life stage (A: Adults = red dots, nests = purple dots; B: Juveniles = green dots; C: Metamorphs = blue dots), LOESS trendlines indicating changes over time. The black line represents the average daily temperature, while the blue ribbon illustrates the daily minimum (*T*_min_) and maximum temperatures (*T*_max_). The bottom panels show the model‐predicted relationships between mean *T*_min6‐0_ and total frogs per survey for each life stage, derived from the top‐ranked Poisson GLMM. Blue lines represent fitted values, and grey shaded areas denote 95% prediction intervals.

Comparing the survey data from the two shelter types, concrete shelters were used more often than the wooden shelters (*β* = −0.14, SE = 0.01, *t* = −13.5; Figure 2). The *C. australis* records consisted of five adults (three under concrete, two under wood), two juveniles (both under wood), and one metamorph (under concrete). *Austrochaperina robusta* records consisted mostly of adults (*n* = 642), followed by juveniles (*n* = 117), metamorphs (*n* = 46), and nests (*n* = 6; Figure 3). Frog numbers followed a seasonal pattern, with the highest number of frogs per survey detected in June to August (Figure 2A) and the predicted monthly probability of detecting *A. robusta* being highest in June to August for both concrete (between 0.80 and 0.92) and wooden (between 0.22 and 0.32) shelters (Figure S3A). Frog numbers under wooden shelters declined over the study period (Figure 2B). When examining different life stages for both shelter types combined, the period with the highest predicted number of adults occurs from May to July (between 0.83 and 0.90), for juveniles in August (0.43), and for metamorphs from June to September (between 0.10 and 0.17; Figure S3B). During the surveys, six *A. robusta* egg clutches (‘nests’) were recorded under the concrete shelters, while no egg clutches were found under the wooden shelters (Figure 3A).

### 3.2 Critical Weather Variables

Sliding‐window analyses found strong evidence that higher frog numbers under concrete shelters were associated with lower minimum temperatures during the 6 days preceding the surveys (*T*_min6‐0_) (Figure S4A–C, Table S1A, ΔAICc = 33). Support was near‐identical across windows of ~7–0 days (Table S2A), indicating that the effect was concentrated in the week before surveys. Sliding‐window scans suggested that the *T*_max_ window (between 30 and 2 days before surveys) might be associated with frog numbers (Figure S4D–F, Table S2B, ΔAICc = 31), with similar support across 18–2 to 13–2 days, consistent with a broad (~2–3 week) pre‐survey window (Table S2B). In addition to associations with temperature, there was evidence that the number of frogs under the shelters was associated with rainfall in the 15 days preceding surveys (Table S2C, ΔAICc = 23), with comparable support across 19–0 days, suggesting that frog numbers declined with increasing rainfall (Table S2C, Figures S1 and S5). There was no evidence to suggest that frog numbers were associated with humidity (Table S1, Figure S3).

Incorporation of *T*_min6‐0_ into the baseline model and rerunning the sliding window analyses for *T*_max_ and rainfall signals showed that *T*_max_ and rainfall did not explain additional variation in frog numbers under concrete shelters. The best‐supported *T*_max_ window failed randomisation (*P*_ΔAICc_ < 0.001, false positive), while the rainfall window showed no support (*P*_ΔAICc_ = 0.94). Therefore, only *T*_min6‐0_ was retained for further analyses.

For wooden shelters, randomisations indicated that the identified best windows were false positives, resulting in no evidence that weather variables were associated with frog numbers under the shelters (Table S1B, Figure S1). This is likely due to the low usage and thus the high number of zero counts in the data.

To assess whether microclimate differences between shelter types influenced frog numbers, we conducted sliding‐window analyses. However, none of the microclimate variables showed support for an association with frog numbers (*P*_ΔAICc_ = 0.75; Figure S2, Table S1C). We conclude that this was due to data limitations (only 12 months of microclimate data from the second season), as including the critical weather variable (*T*_min_) from our full dataset also did not receive support.

### 3.3 Model Selection

Model selection evaluated 106,497 candidate models (see summary in Table 3; top 100 best supported models in Table S3). Frog numbers were higher under concrete shelters (*n* = 610 records of frogs) compared to wooden shelters (*n* = 195 records; Figure 2). Including shelter type as a predictor in the model was strongly supported, with shelter type included in 46% of models (ΔAICc = 243; Table 3). However, the effect of shelter type varied with life stage. Juveniles were less frequently under wooden shelters than adults (*β* = −0.19 ± 0.01), whereas metamorphs were more frequently recorded under wooden than under concrete shelters (*β* = 0.38 ± 0.02). This life stage × shelter type interaction received strong model support (Table 3, model 1 vs. model 10). Overall, adults were recorded in shelters in greater numbers compared to juveniles and metamorphs (ΔAICc = 784.2; Table 3, model 1 vs. model 13; Figure 3).

**TABLE 3:** Summary of model selection results assessing the influence of environmental and habitat variables on monthly frog numbers in shelters between February 2023 and January 2025. Shown are standardised coefficients (±SE) for variables included in the best‐supported models where each predictor was present, based on AICc. These models were used to determine the relative importance of individual predictors. Variables deemed important in the best‐supported model are those with 95% confidence intervals that do not overlap zero; these are shown in italics. Models were ranked by ΔAICc values. ‘n.a.’ denotes variables not included in a given model, while ‘+’ indicates that the variable was included. Predictors that did not appear in any of the best‐supported models are not shown to maintain table clarity; see Table 1 for full list.

Model	Intercept	Vegetation	Distance shelters	Social insects	Hard bodied	Potential predators	Soft bodied	Life stage	*T*_min_	Shelter type	Life stage × shelter type	*T*_min_ × shelter type	df	AICc	ΔAICc
1	*−0.93* ± *0.02*	0.27 ± 0.02	−0.45 ± 0.02	−0.35 ± 0.03	−0.24 ± 0.02	−0.22 ± 0.02	−0.47 ± 0.03	+	*−0.17* ± *0.01*	+	+	NA	14	3708.29	0.00
2	−0.94	0.27	−0.46	−0.38	−0.25	NA	−0.48	+	−0.17	+	+	NA	13	3708.35	0.06
3	−0.94	0.27	−0.46	−0.37	NA	−0.23	−0.51	+	−0.18	+	+	NA	13	3708.50	0.21
4	−0.93	0.27	−0.45	−0.34	−0.24	−0.22	−0.34	+	−0.20	+	+	+	15	3708.78	0.49
6	−0.94	0.27	−0.45	NA	−0.25	−0.24	−0.47	+	−0.18	+	+	NA	13	3709.09	0.80
7	−0.93	0.27	−0.45	−0.35	−0.27	−0.22	NA	+	−0.17	+	+	NA	13	3709.78	1.48
8	−0.93	NA	−0.45	−0.35	−0.24	−0.22	−0.47	+	−0.17	+	+	NA	13	3710.52	2.23
9	−0.92	0.28	NA	−0.35	−0.24	−0.22	−0.47	+	−0.17	+	+	NA	13	3716.22	7.93
10	−0.95	0.27	−0.45	−0.35	−0.24	−0.22	−0.47	+	−0.17	+	NA	NA	12	3729.53	21.23
11	−0.89	0.28	−0.45	−0.49	−0.34	−0.26	−0.51	+	NA	+	+	NA	13	3730.56	22.27
12	−1.40	0.27	−0.45	−0.22	−0.40	−0.07	−0.51	+	−0.17	NA	NA	NA	11	3953.41	245.1
13	−1.85	0.27	−0.45	−0.35	−0.24	−0.22	−0.47	NA	−0.17	+	NA	NA	10	4492.48	784.2

Among environmental and weather predictors, frog numbers were negatively associated with *T*_min6‐0_, such that higher frog numbers were recorded following lower minimum temperatures. This relationship was particularly pronounced for concrete shelters (*β* = −0.05 ± 0.01), and less so for wooden shelters (*β* = 0.03 ± 0.01; Figure 2C,D). However, the difference in the association with *T*_min6‐0_ between shelter types only received weak model support (Table 3, model 1 vs. model 4). Higher frog numbers were observed when concrete and wooden shelters were placed close together, and this association was strongly supported (Table 3, model 1 vs. model 9). Similarly, higher frog numbers were observed with higher undergrowth cover (Table 3, model 1 vs. model 8). However, there was no evidence that other environmental predictors (i.e., canopy cover, elevation, leaf litter, woody debris, and distance to nearby objects) were associated with frog numbers, as adding these variables to the top model reduced model support (ΔAICc > +1.2, Table S3). There was some evidence that the presence of invertebrates was negatively associated with frog numbers (Table 3, model 1 vs. models 2, 3, 6, 7). This suggests that frogs and invertebrates may occasionally avoid shelters when either is present. The best supported model explained a substantial proportion of the variation in frog numbers ($R_{m}^{2}$ = 0.44, $R_{c}^{2}$ = 0.52; lognormal approximation), indicating that almost half of the variation of the frog numbers recorded under the shelter was determined by environmental variables and habitat characteristics.

### 3.4 Microclimate

**FIGURE 4:**
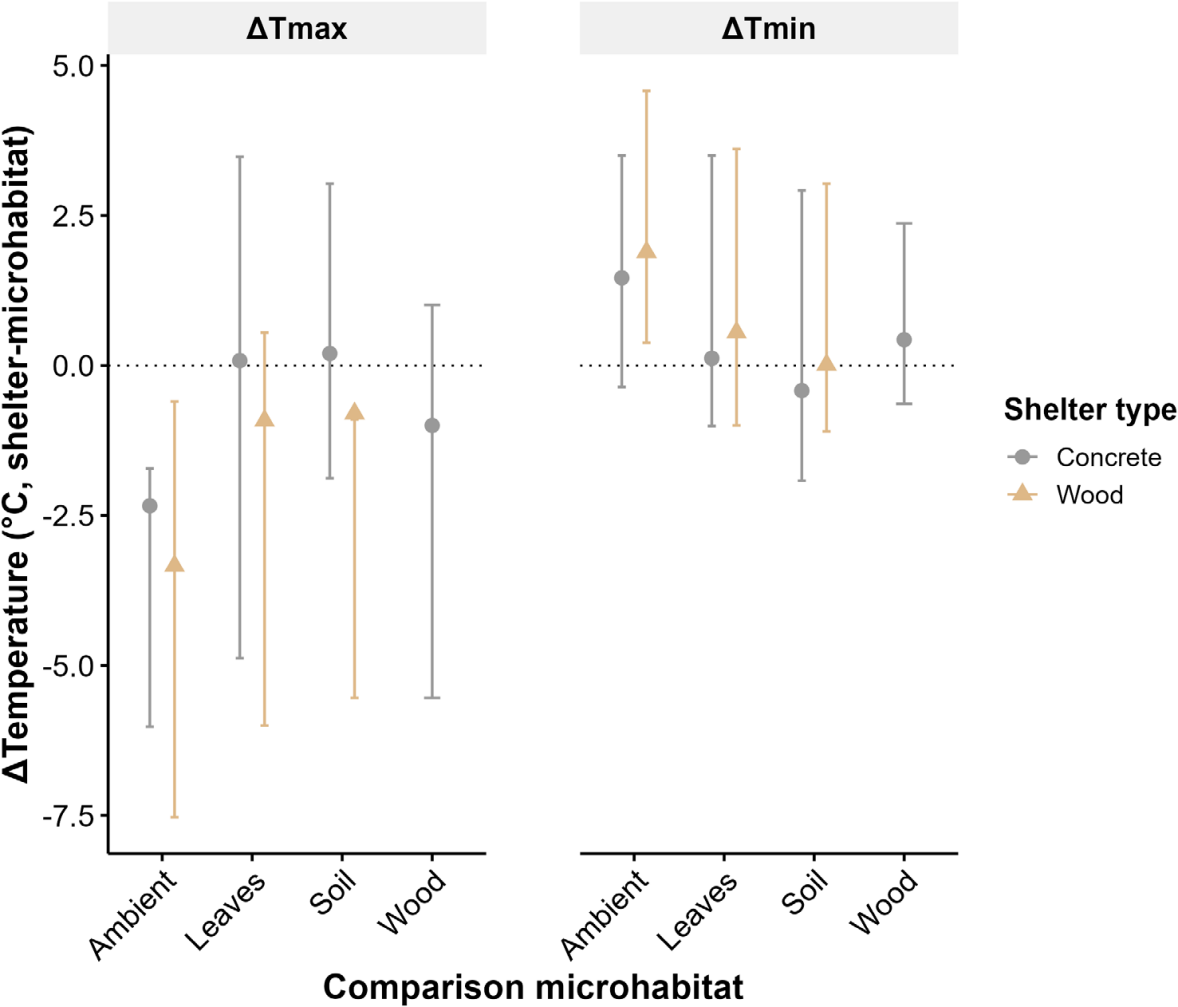
Differences in maximum (Δ*T*_max_) and minimum (Δ*T*_min_) microclimate temperatures between artificial shelters (concrete and wood) and between shelters and surrounding microhabitats (ambient, leaves, soil). Points represent mean temperature differences (±range) across the 2024 study period. Positive values indicate warmer temperatures inside shelters relative to the comparison microhabitat, while negative values indicate cooler temperatures.

Microclimate data showed that concrete shelters were on average warmer than wooden shelters (mean difference 1.00°C, range: −1.01°C to +5.54°C) and the other microhabitats (soil and leaves) during the day, but were cooler compared to ambient daytime temperatures (Figure 4, Figure S6). The concrete shelters were also warmer on average than leaves and ambient temperature at night (*T*_min_), while being cooler than wooden shelters and soil (Figure 4). In contrast, wooden shelters provided cooler maximum temperatures but higher minimum temperatures (*T*_min_) across the year. Wood was cooler than ambient air during the day, and cooler than concrete (−1.00°C, range: −5.54°C to +1.01°C), soil, and leaves (Figure 4). At night, wood retained higher minimum temperatures compared to the ambient and all other microhabitats (Figure 4, Figures S6 and S7). When separating the peak period of frog shelter use (dry season; June–August) from the low‐use period (wet season; November–January), clear seasonal differences emerged in the thermal contrast between shelter types. During the wet season, concrete shelters were on average 0.84°C ± 0.36°C (SD) warmer than wooden shelters during the day and 0.46°C ± 0.15°C warmer at night, whereas during the dry season concrete shelters were only marginally warmer during the day (0.17°C ± 0.52°C) and were cooler than wooden shelters at night (−0.23°C ± 0.25°C; Figure S7). However, the sliding window analyses showed no evidence that any of the microclimate variables were associated with frog numbers (*P*_ΔAICc_ = 0.75; Figure S2, Table S1C). We conclude that this was due to data limitations (only 12 months of microclimate data from the second season), because including the weather station *T*_min6‐0_ also did not receive support in this reduced dataset.

## 4 Discussion

Across 2 years in upland tropical rainforest, *A. robusta* was observed 801 times under artificial shelters, demonstrating frequent use by this small, fossorial, terrestrial‐breeding frog species across all life stages, from eggs to adults. In contrast, the other microhylid frog species in the area, *C. australis* , was rarely recorded under the shelters (eight observations). This limited use is consistent with its scansorial (i.e., partly arboreal) ecology, with individuals known to seek refuge and breed in elevated situations rather than exclusively in ground‐level shelters (Hoskin 2004, 2013; Zweifel 1985). *Austrochaperina robusta* consistently used concrete over wooden shelters, particularly during cooler and drier periods. This pattern aligns with the greater thermal buffering provided by concrete shelters and highlights temperature‐mediated refuge selection as a key mechanism shaping shelter use in this species.

Our study revealed that *A. robusta* used the concrete shelters more than wooden shelters, consistent across survey periods and all life stages except metamorphs (which were more common under the wooden shelters). Additionally, all observed egg clutches were under concrete shelters, indicating that they provide more suitable breeding microhabitats than wooden shelters. This is likely due to their greater structural stability and warmer microclimatic conditions, which may promote embryonic development and reduce exposure of eggs to physical disturbance. Selection of warmer oviposition sites is consistent with studies of terrestrial‐breeding amphibians, where temperature influences embryonic development and site choice (Touchon and Worley 2015). Concrete, with greater thermal mass and conductivity, absorbs and stores more daytime heat than wood (Dodoo et al. 2012), resulting in higher maximum temperatures beneath shelters, particularly in cooler months. At night, concrete cooled faster, whereas wooden shelters maintained more stable conditions. This stability may partly reflect the fact that some wooden shelters were larger, providing greater thermal buffering. Despite cooler nocturnal air temperatures, heat stored in the concrete was released, and frogs likely gained warmth conductively from the surface. This retained heat could be valuable during cold periods and may explain the stronger use of concrete shelters by *A. robusta* in cooler months. Although chytrid fungus, *Batrachochytrium dendrobatidis*, is uncommon in microhylids (Hauselberger and Alford 2012), these warmer refuges may still raise body temperatures in a beneficial way to suppress infection, as observed in *Litoria aurea* (Waddle et al. 2024).

Shelter use by *A. robusta* varied seasonally, with the highest numbers recorded during the cooler and drier months (June–July) and the lowest during the hot, wet period (December–January). This seasonal pattern was closely linked to temperature, especially in concrete shelters, with frog numbers increasing under cooler conditions a week before sampling, and declining during warmer, wetter periods. These results indicate that cooler conditions promote increased use of artificial shelters, particularly concrete shelters, by *A. robusta* , likely because they provide thermally buffered microhabitats during periods when other microhabitat temperatures are suboptimal. Although the thermal preference of *A. robusta* is currently unknown, a closely related species, *Cophixalus australis* , also recorded using the artificial shelters in this study, has a preferred body temperature of approximately 24.7°C (Merino‐Viteri 2018), suggesting that seasonal shelter use may reflect behavioural thermoregulation relative to species‐specific thermal optima. Metamorphs were recorded only between June and October, consistent with their development from eggs laid during the warm wet season and their growth to metamorph size by mid‐year. Similarly, most nesting activity (five of six nests) occurred during the known peak breeding season, with one additional nest observed in September following a rain event during an otherwise dry period. Although the temperature effect on frog numbers was observed across all life stages, it was most pronounced in adults, possibly reflecting differences in thermal preferences or activity among life stages, as shown in other direct‐developing species (Scheffers et al. 2014).

Although overall frog numbers appeared to decline slightly over the 2‐year study, longer‐term monitoring will be required to determine whether this reflects natural interannual variation driven by climatic conditions or other ecological factors. Shelter effectiveness may also change through time as surrounding habitat structure is altered (e.g., by tree falls) or as shelters degrade, particularly wooden shelters that decompose rapidly in tropical rainforest environments. These factors highlight the importance of considering shelter longevity and habitat dynamics when interpreting temporal patterns of shelter use. We quantified frog numbers exclusively under artificial shelters and did not survey other (natural) refuges. While natural refuges such as logs, soil, leaf litter, and rocks are readily identifiable, frogs within these microhabitats, particularly in soil and leaves, are often undetectable, leading to a high risk of missed detections. Consequently, our results describe patterns of artificial shelter use rather than overall habitat use or population abundance.

Although moisture is a well‐recognised driver of amphibian activity and refuge use, with many species becoming active and breeding during wet periods and seeking more permanent shelters during dry conditions (Todd et al. 2011; Wells 2010), our results did not indicate that rainfall predicted shelter use by *A. robusta* well. This is probably due to the high collinearity between rainfall and temperature, as roughly 84% of annual rainfall occurs during the hottest months in the Wet Tropics mountains (Congdon and Herbohn 1993). Therefore, temperature captured most of the environmental variation relevant to shelter use. Because *A. robusta* has a small home range (Groffen et al. 2025) and does not consistently return to the same daytime refuge (Groffen et al. 2025; Merino‐Viteri 2018), individuals can readily shift among nearby microhabitats. Under warmer and wetter conditions, alternative refuges such as leaf litter and moist soil may become more favourable by providing more humid environments that facilitate cutaneous respiration and activity. The structure of understorey vegetation likely contributes to these dynamics by enhancing shade and moisture retention (De Frenne et al. 2019; Fu et al. 2025; Sayer 2006). Understorey vegetation may also contribute by providing leaf litter and prey (Uetz 1979), and by influencing soil composition (Ritchie and Dolling 1985; Yan et al. 1996).

While thermal conditions and habitat characteristics have been shown to play a role in shelter selection, other ecological factors, such as disturbance and threats, might also appear influential. Large invertebrates like centipedes (Forti et al. 2007; pers. obs.), earthworms (pers. obs.), and spiders (Fulgence et al. 2021; Menin et al. 2005) may prey on the frog or their eggs and even non‐lethal interactions may cause disturbances (Groffen and Hoskin 2024) that increase energetic costs and reduce shelter attractiveness. While ants can serve as prey for the microhylid frogs (Hoskin 2004), they were typically present as a colony rather than solitary foragers, making them more of a potential threat than a food source. Invertebrates were found in both shelter types, but groups such as hard‐bodied insects were more common in wooden shelters. In the tropical rainforest environment wooden shelters decompose rapidly and invertebrate species feed on the decaying wood or occupy the hollows that develop during decomposition. Earthworm castings often blocked entrances of concrete shelters, requiring manual removal during shelter checks. Taken together, invertebrate presence appears to be a subtle but consequential ecological factor that may influence which refuges frogs choose to occupy.

This study demonstrates that artificial shelters provide a valuable, minimally invasive tool for monitoring and potentially conserving cryptic, fossorial frogs such as *A. robusta* . For this species, shelter use was closely linked to microclimatic conditions, with thermally buffered (concrete) shelters supporting higher frog numbers and providing suitable sites for breeding. Although shelter use was dominated by this ground‐dwelling species, the occasional use of shelters by the scansorial frog *C. australis* suggests broader applicability across species. Our results suggest that concrete shelters would be used by other similar‐sized microhylid species elsewhere in the Wet Tropics and by hundreds of other small, terrestrial frog species, globally. Further research could test preference for varied chamber and entrance/exit tunnel sizes by different size species. Overall, artificial shelters, especially when designed to match species‐specific ecology, monitoring approaches (e.g., camera‐based methods; Groffen and Hoskin 2024), and habitat requirements hold strong potential as a scalable conservation and monitoring tool under increasing climate variability.

## Author Contributions

**Jordy Groffen:** conceptualization (lead), data curation (lead), formal analysis (lead), funding acquisition (lead), investigation (lead), methodology (lead), project administration (lead), resources (lead), visualization (lead), writing – original draft (lead). **Lyanne Brouwer:** formal analysis (supporting), methodology (supporting), supervision (equal), validation (supporting), visualization (supporting), writing – review and editing (equal). **Myles H. M. Menz:** supervision (equal), writing – review and editing (equal). **Conrad J. Hoskin:** conceptualization (supporting), formal analysis (supporting), methodology (supporting), supervision (equal), visualization (supporting), writing – review and editing (equal).

## Funding

This work was supported by Holsworth Wildlife Research Endowment Grant (Ecological Society of Australia Ltd), the Skyrail Rainforest Research Fund (Skyrail Rainforest Foundation), and the Ric Nattrass Research Grant (Queensland Frog Society Inc).

## Ethics Statement

This research was conducted under James Cook University Animal Ethics approval A2857 and Queensland Government scientific research permits P‐PTUKI‐100306983‐1 and P‐PTUKI‐100306983.

## Conflicts of Interest

The authors declare no conflicts of interest.

## Supporting information


**Data S1:** ece373215‐sup‐0003‐Supinfo.docx.

## Data Availability

All the required data are uploaded as Supporting Information.
