# Supplementary material for: Artificial Shelters as a Monitoring and Conservation Tool for Terrestrial Breeding Frogs
Source: Ecol Evol. 2026 Mar 12;16(3):e73215. doi: 10.1002/ece3.73215 (PMC13093504; doi:10.1002/ece3.73215)

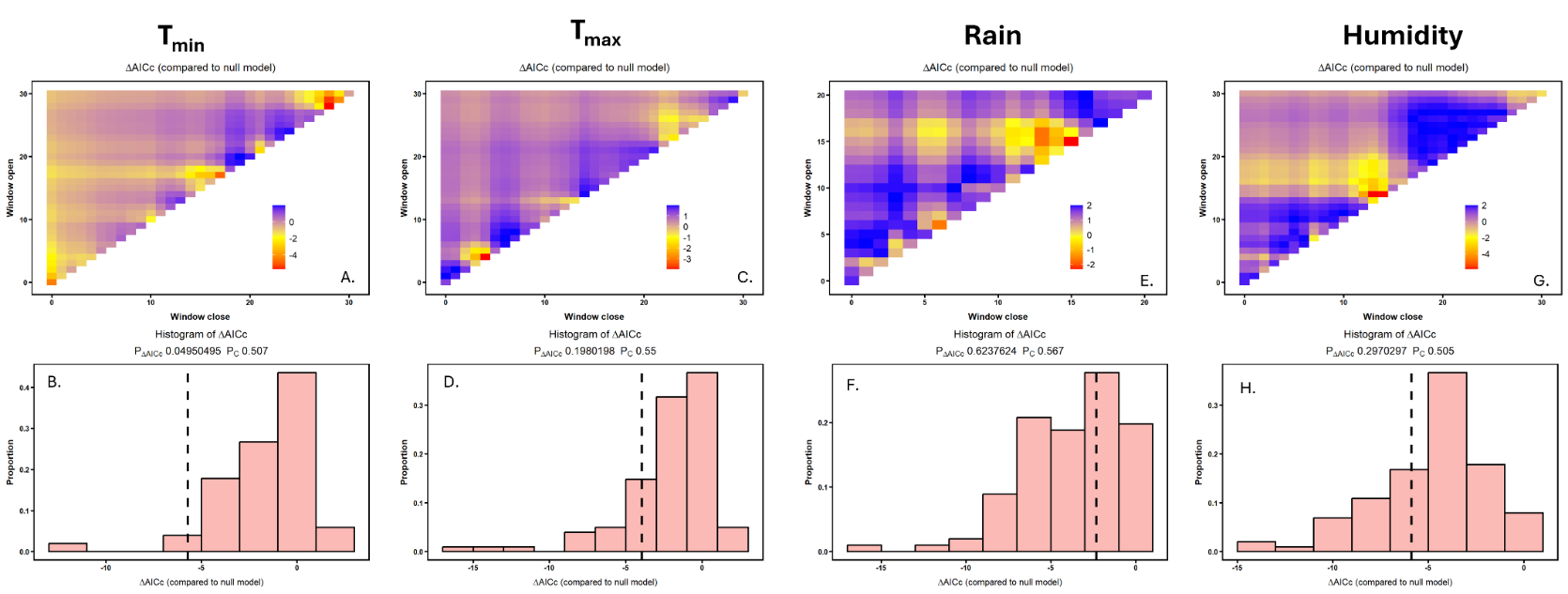


**Figure S1**. Results from *climwin* analyses testing the minimum temperature (T_min_; A–B), maximum temperature (T_max_; C–D), and rainfall (E–F) as predictors of frog occupancy in wooden shelters. None of the predictors shows a clear signal. (A, C, E) Heatmaps show the relative support (ΔAICc values) for climatic windows opening (y-axis) and closing (x-axis) in days before each survey. Warmer colours indicate stronger model support (lower ΔAICc compared to the null model). (B, D, F) Histograms show the distribution of ΔAICc values from 101 randomised datasets compared to the observed model (dotted line).


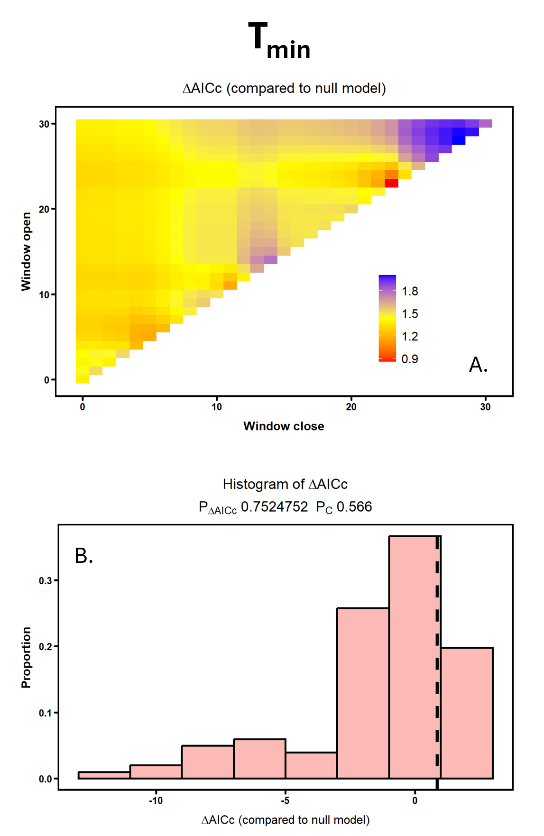

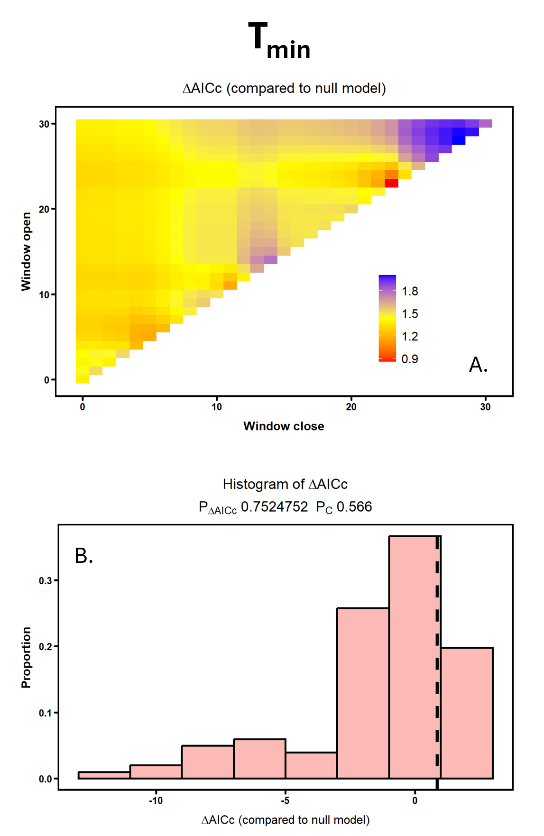


**Figure S2.** Results from *climwin* analyses testing minimum temperature (T_min_) under the concrete shelters (microclimate; A–B), as predictors of frog occupancy in concrete shelters. We chose T_min_ and concrete shelters because the ambient T_min_ in the whole data set gave a strong climate signal. (A) Heatmap shows the relative support (ΔAICc values) for climatic windows opening (y-axis) and closing (x-axis) in days before each survey. Warmer colours indicate stronger model support (lower ΔAICc compared to the null model). (B) Histograms show the distribution of ΔAICc values from 101 randomised datasets compared to the observed model (dotted line).


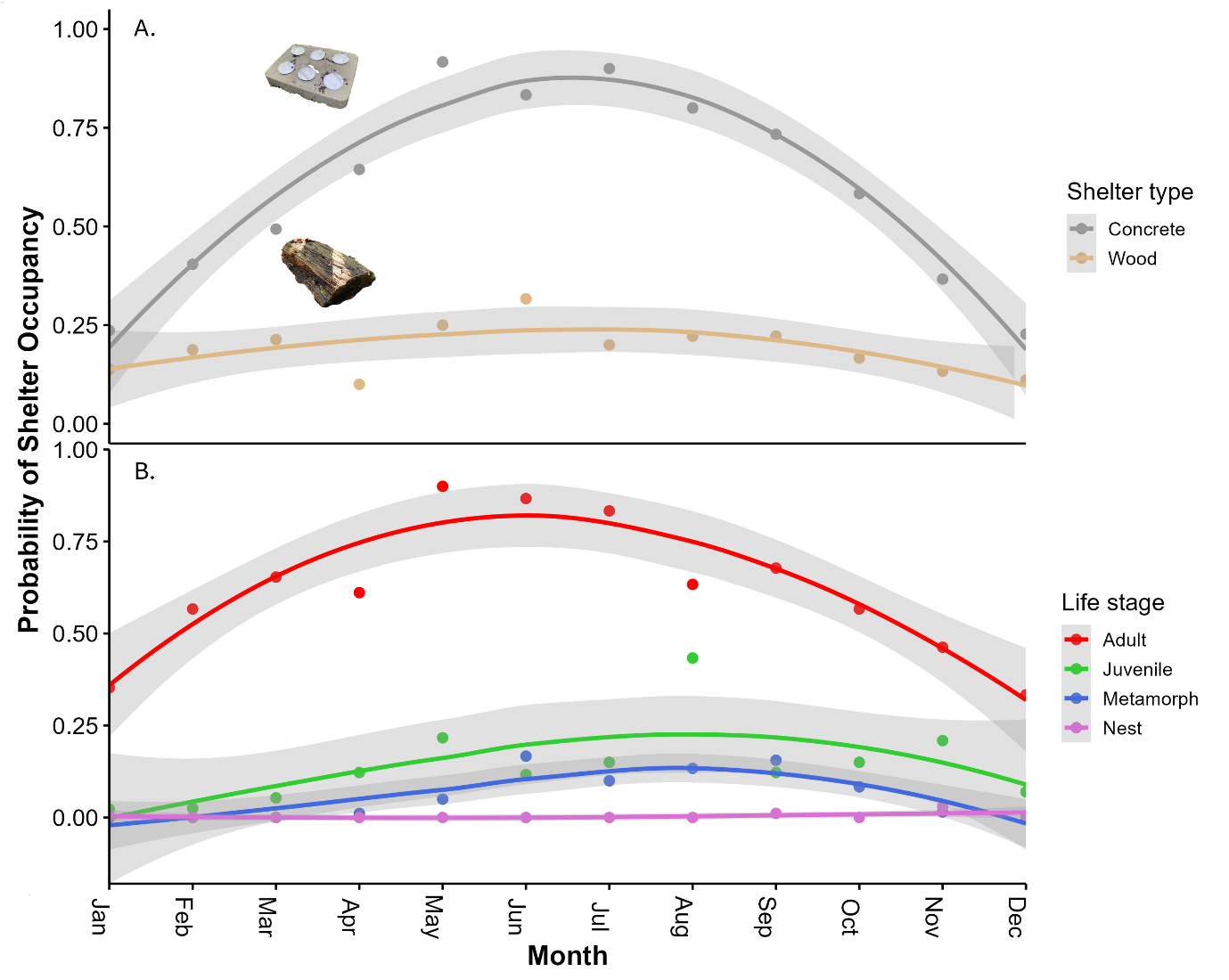


**Figure S3.** Panel A. shows the predicted probability of total number of *Austrochaperina robusta* individuals recorded per shelter type (concrete = grey dots and wood = brown dots), with trendlines indicating changes over time, along with the 95% confidence interval (dark grey). Panel B. presents the predicted probability of total number of *A. robusta* of different life stages (shelter types combined): adults (red dots), juveniles (green dots), metamorphs (blue dots), and nets (purple dots). Predictions of monthly frog occupancy per life stage and shelter type were generated using the predict() function with type = "response" to obtain values on the original count scale).


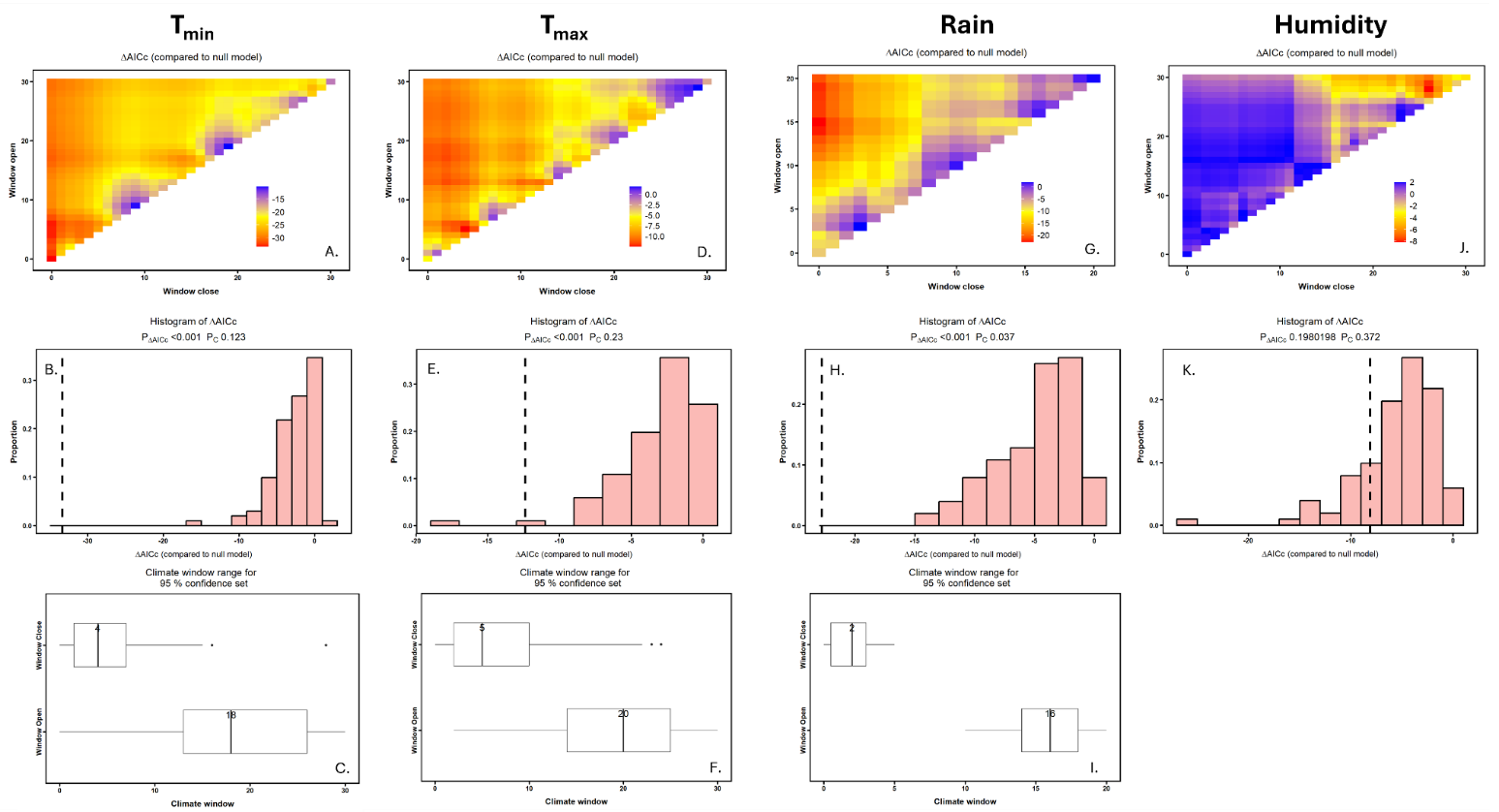


**Figure S4.** Results from climwin analyses testing the minimum temperature (T_min_; A–C), maximum temperature (Tmax; D–F), rainfall (G–I), and humidity (J–K) as predictors of frog occupancy in concrete shelters. (A, D, G) Heatmaps show the relative support (ΔAICc values) for climatic windows opening (y-axis) and closing (x-axis) in days before each survey. Warmer colours indicate stronger model support (lower ΔAICc compared to the null model). (B, E, H) Histograms show the distribution of ΔAICc values from 101 randomised datasets compared to the observed model (dotted line). (C, F, I) Boxplots show the 95% confidence range for the best-supported climate windows. For T_min_ (C), the effect window spans approximately 18–4 days pre-survey, for T_max_ (F) 20–5 days pre-survey, and rain (L.) 16–2 days pre-survey.


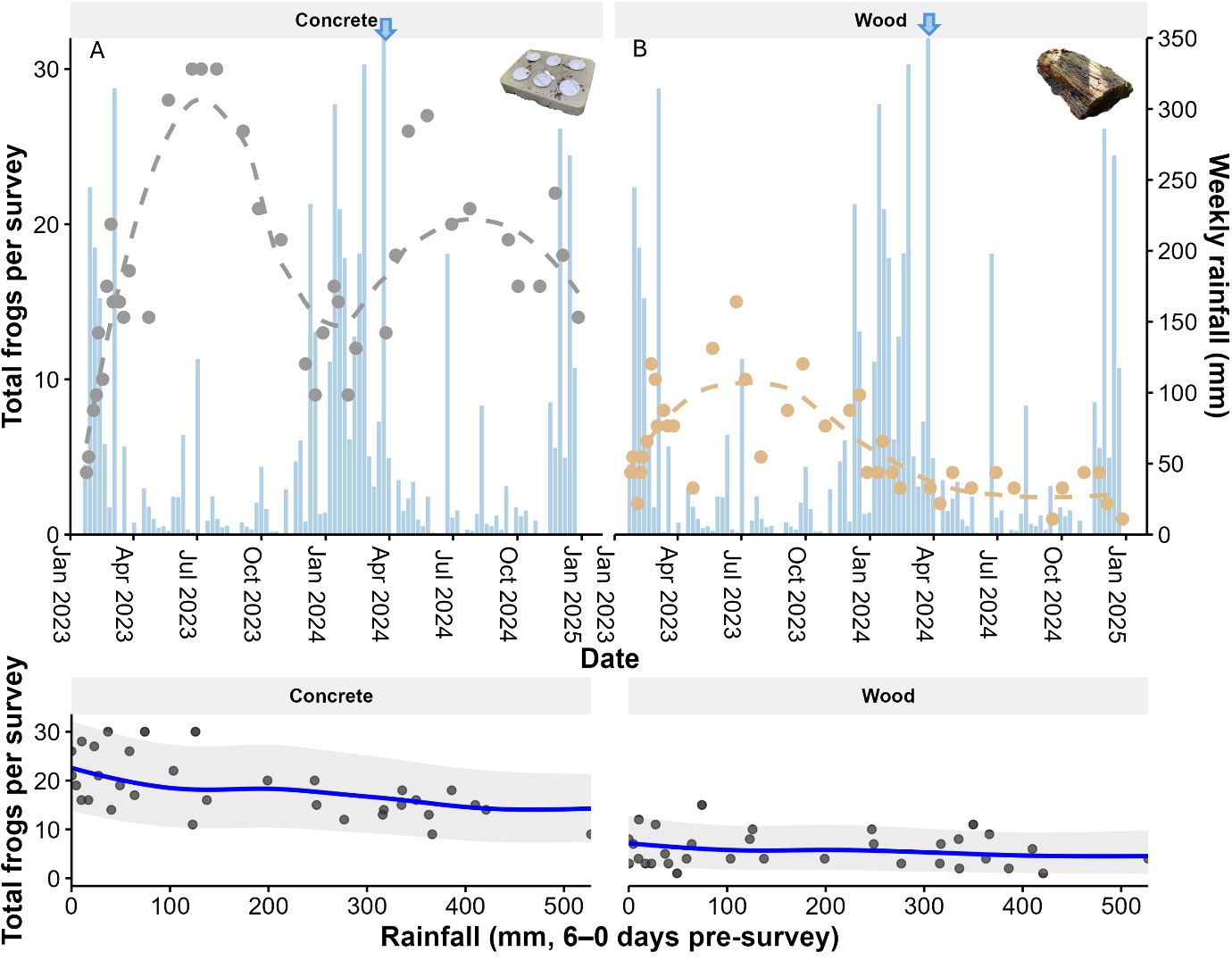


**Figure S5.** The top panels display the total number of *Austrochaperina robusta* individuals recorded per shelter during occupancy surveys, separated by shelter type (A. concrete: grey dots, n = 610; B. wood: brown dots, n = 195), with dashed LOESS trendlines indicating changes over time. The blue bars represent the average weekly rainfall . The bottom panels show the model-predicted relationships between mean rainfall (15–2 days pre-survey) and total frogs per survey for each shelter type. Blue lines represent fitted values, and grey shaded areas denote 95% prediction intervals.

**
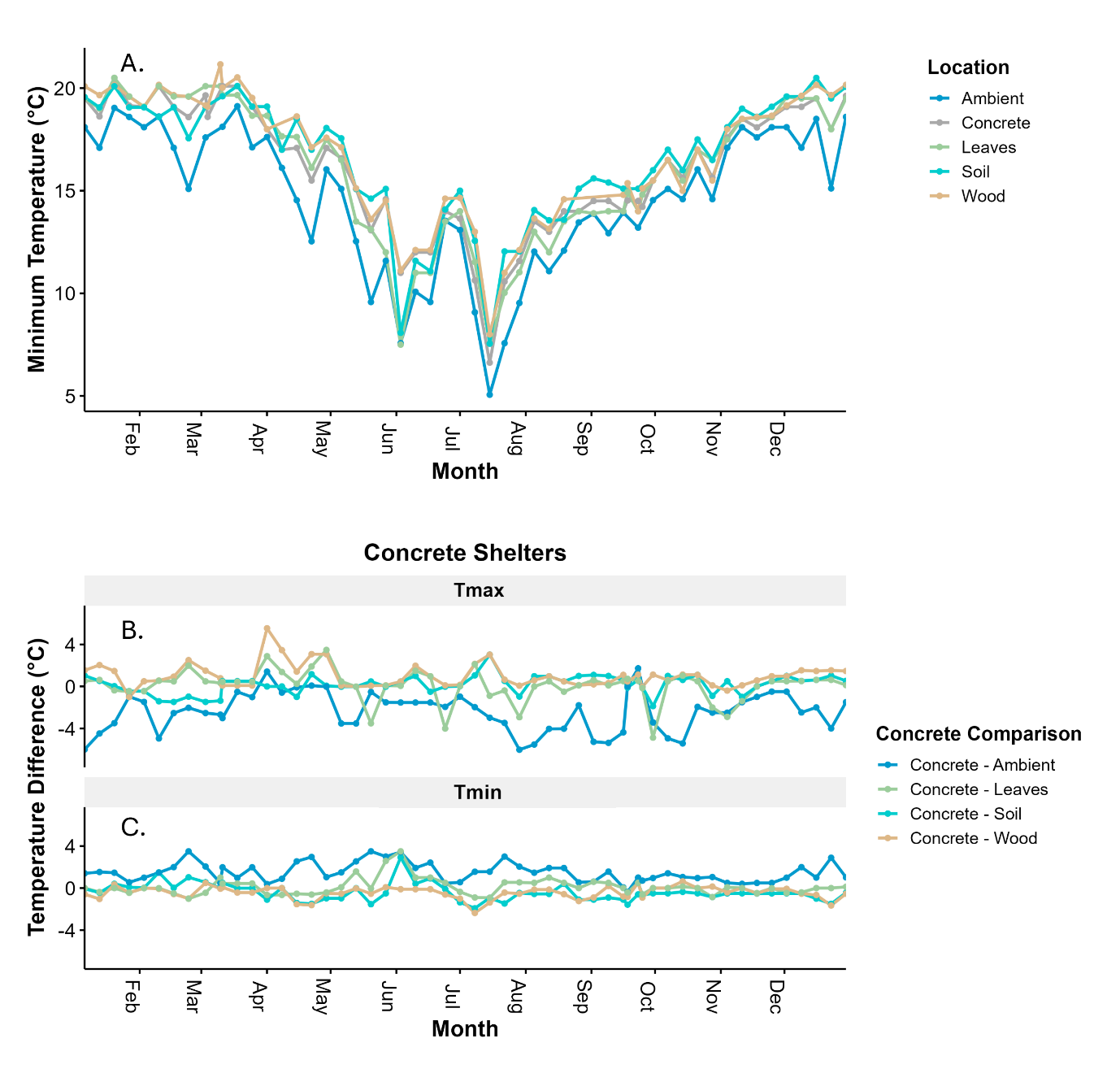
**

**Figure S6.** Microhabitat temperature comparison (January to December 2024). (A) Weekly minimum temperatures measured in five microhabitats: ambient air (1.2 m above ground), under concrete and wooden shelters, beneath leaf litter, and in soil at 4 cm depth (January–December 2024). (B–C) Temperature differences between concrete shelters and other microhabitats (ambient, leaf litter, soil, wood) across the year: (B) maximum and (C) minimum. Positive values indicate temperatures under concrete are higher than in the other microhabitat (ambient, leaf litter, soil, or wood).


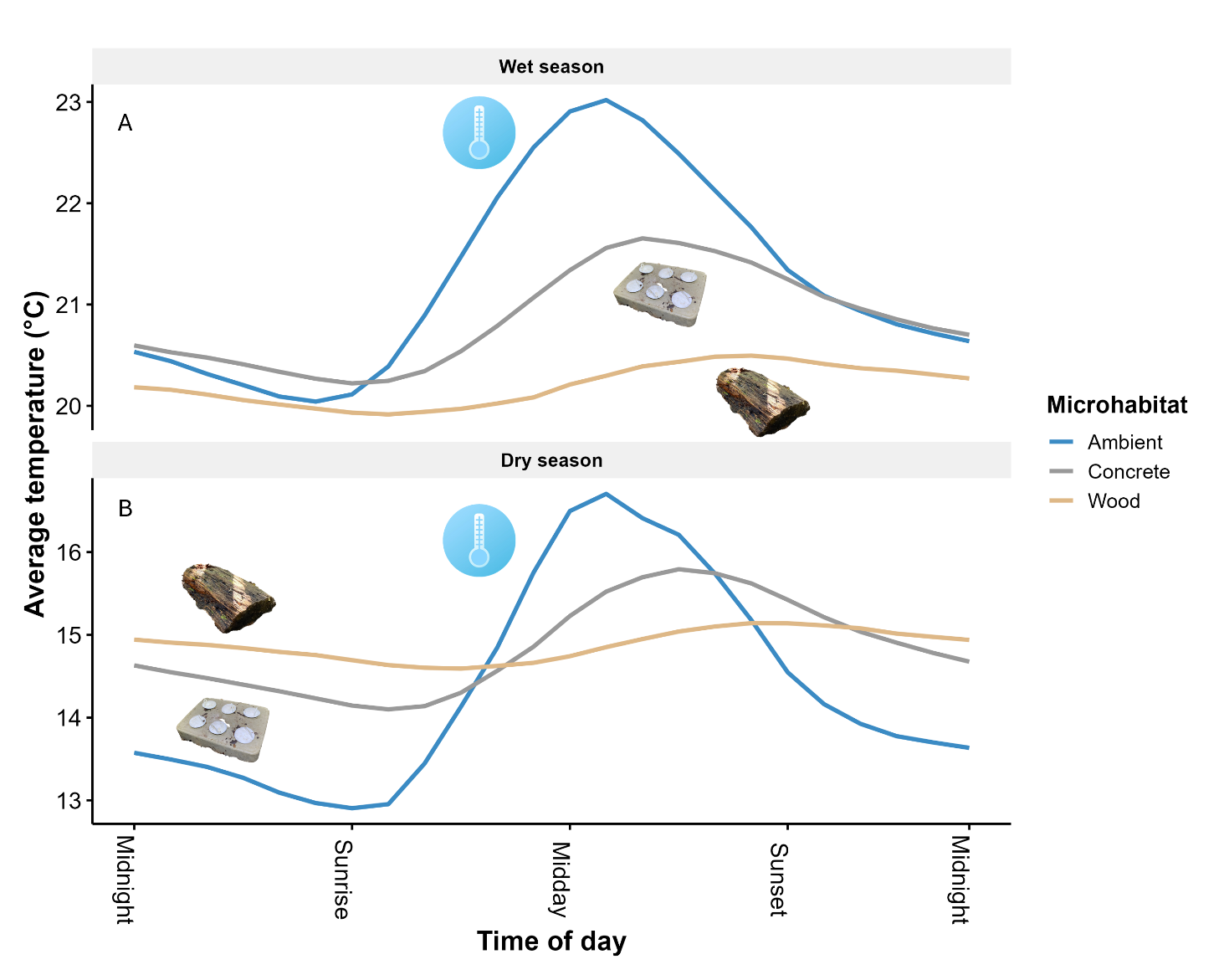


Figure S7. Mean diel temperature profiles of ambient air and artificial shelter microhabitats (concrete and wood) during the wet season (A; November–January) and dry season (B; June–August). Lines show hourly mean temperatures averaged across sampling days. Supplementary

**Table S1.** Results of the critical climate window analyses for predictors influencing frog occupancy in concrete and wooden shelters. The table shows the best-supported opening and closing windows (in days prior to surveys), model coefficients (β ± SE), test statistics (Z, p), and direction of the relationship between climatic variables and occupancy. For concrete shelters, occupancy decreased with warmer minimum (T_min_) and maximum (T_max_) temperatures and with higher rainfall, whereas humidity showed a false-positive signal that did not survive the randomisation test. All climatic variables for wooden shelters failed to survive randomisation, indicating no consistent signal. A separate analysis of T_min_ measured directly under concrete shelters also showed no significant effect.

| 1. *Concrete shelters* | | |  |  | | | |  | |  | |  |  |  |  |
| --- | --- | --- | --- | --- | --- | --- | --- | --- | --- | --- | --- | --- | --- | --- | --- |
| **Predictor** | **Window opens** | **Window closes** | | | **β** | **SE** | **Z** | | **P_ΔAICc_** | | Pc | | | | |
| T_min_ (mean) | 6 | 0 | | | -0.06 | 0.01 | -6.06 | | **< 0.001** | | 0.12 | | | | |
| T_max_ (mean) | 30 | 2 | | | -0.08 | 0.01 | -5.82 | | **< 0.001** | | 0.23 | | | | |
| Rain (sum) | 15 | 2 | | | -0.001 | 0.0003 | -4.87 | | **< 0.001** | | 0.04 | | | | |
| Humidity (mean) | False positive |  | | | 0.18 | 0.06 | 2.7 | | 0.20 | | 0.37 | | | | |
|  |  | |  |  | | | |  | |  | |  |  |  |  |
|  |  | |  |  | | | |  | |  | |  |  |  |  |
| 1. *Wooden shelters* | | |  |  | | | |  | |  | |  |  |  |  |
| **Predictor** | **Window opens** | **Window closes** | | | **β** | **SE** | **Z** | | **P_ΔAICc_** | | Pc | | | | |
| T_min_ (mean) | False positive |  | | | -0.05 | 0.02 | -2.82 | | **0.05** | | 0.51 | | | | |
| T_max_ (mean) | False positive |  | | | -0.05 | 0.02 | -2.42 | | 0.20 | | 0.55 | | | | |
| Rain (sum) | False positive |  | | | -0.017 | 0.009 | -1.9 | | 0.62 | | 0.57 | | | | |
| Humidity (mean) | False positive |  | | | 0.18 | 0.06 | -3.04 | | 0.30 | | 0.51 | | | | |
|  |  |  | | |  |  |  | |  | |  | | | | |
|  |  | |  |  | | | |  | |  | |  |  |  |  |
| 1. *T_min_ microclimate under concrete shelter* | | |  |  | | | |  | |  | |  |  |  |  |
| **Predictor** | **Window opens** | **Window closes** | | | **β** | **SE** | **Z** | | **P_ΔAICc_** | | Pc | | | | |
| T_min_ (mean) | False positive |  | | | -0.03 | 0.04 | -1.07 | | 0.75 | | 0.57 | | | | |

**Table S2.** Top 10 sliding-window results for minimum daily temperature (Tmin), maximum daily temperature (Tmax), and daily rainfall on frog numbers in concrete shelters, using mean values for temperatures and summed rainfall, with a linear (lin) function across 0–30 days prior to surveys.

| **Predictor** | **Model** | **ΔAICc** | **WindowOpen** | **WindowClose** | **Climate±SE** |
| --- | --- | --- | --- | --- | --- |
| 1. **T_min_** |  |  |  |  |  |
| T_min_ | 28.00 | -33.12 | 6 | 0 | -0.06 ± 0.009 |
| T_min_ | 15.00 | -33.02 | 4 | 0 | -0.05 ± 0.009 |
| T_min_ | 21.00 | -33.01 | 5 | 0 | -0.06 ± 0.009 |
| T_min_ | 3.00 | -32.45 | 1 | 0 | -0.05 ± 0.009 |
| T_min_ | 10.00 | -32.32 | 3 | 0 | -0.06 ± 0.01 |
| T_min_ | 36.00 | -31.74 | 7 | 0 | -0.06 ± 0.01 |
| T_min_ | 6.00 | -31.63 | 2 | 0 | -0.05 ± 0.009 |
| T_min_ | 27.00 | -31.44 | 6 | 1 | -0.06 ± 0.01 |
| T_min_ | 20.00 | -31.12 | 5 | 1 | -0.05 ± 0.009 |
| 1. **T_max_** |  |  |  |  |  |
| T_max_ | 494 | -31.03 | 30 | 2 | -0.08 ± 0.013 |
| T_max_ | 169 | -30.97 | 17 | 2 | -0.07 ± 0.012 |
| T_max_ | 188 | -30.84 | 18 | 2 | -0.07 ± 0.012 |
| T_max_ | 208 | -30.80 | 19 | 2 | -0.07 ± 0.012 |
| T_max_ | 496 | -30.77 | 30 | 0 | -0.08 ± 0.013 |
| T_max_ | 433 | -30.71 | 28 | 2 | -0.07 ± 0.013 |
| T_max_ | 495 | -30.67 | 30 | 1 | -0.08 ± 0.013 |
| T_max_ | 404 | -30.63 | 27 | 2 | -0.07 ± 0.013 |
| T_max_ | 171 | -30.59 | 17 | 0 | -0.07 ± 0.012 |
| T_max_ | 210 | -30.56 | 19 | 0 | -0.07 ± 0.012 |
| 1. **Rainfall** |  |  |  |  |  |
| Rainfall | 136 | -22.78 | 15 | 0 | -0.001 ± 0.002 |
| Rainfall | 120 | -22.51 | 14 | 0 | -0.001 ± 0.003 |
| Rainfall | 210 | -22.21 | 19 | 0 | -0.001 ± 0.004 |
| Rainfall | 190 | -21.94 | 18 | 0 | -0.001 ± 0.005 |
| Rainfall | 171 | -21.76 | 17 | 0 | -0.001 ± 0.006 |
| Rainfall | 153 | -21.76 | 16 | 0 | -0.001 ± 0.007 |
| Rainfall | 253 | -21.54 | 21 | 0 | -0.001 ± 0.008 |
| Rainfall | 276 | -21.46 | 22 | 0 | -0.001 ± 0.009 |
| Rainfall | 135 | -21.18 | 15 | 1 | -0.001 ± 0.010 |
| Rainfall | 105 | -21.04 | 13 | 0 | -0.001 ± 0.011 |


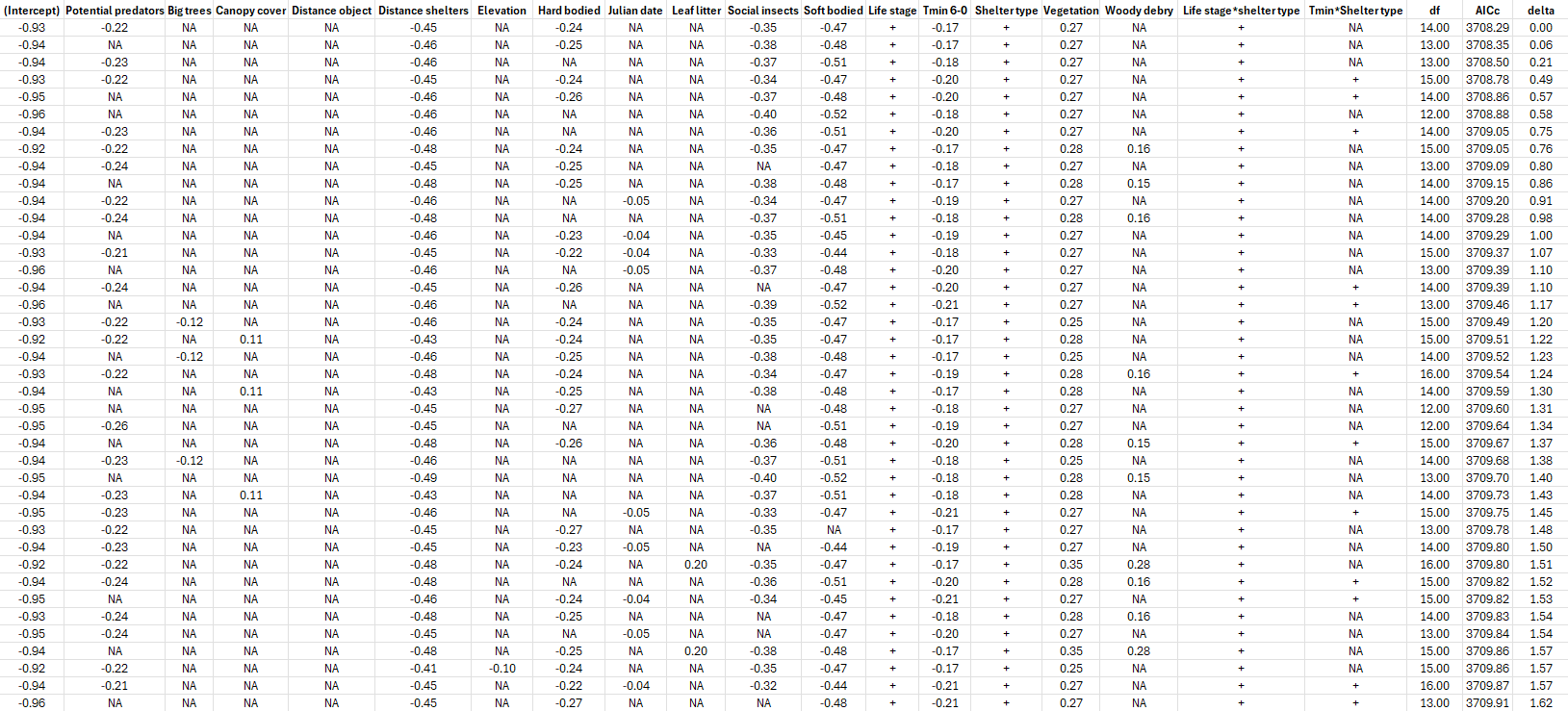
Table S3. Summary of the top 100 candidate models assessing the influence of environmental and habitat variables on monthly frog numbers in shelters between February 2023 and January 2025. Models were ranked by ΔAICc values. These models were used to assess the relative importance of individual predictors across the candidate set. Variables were considered influential when their 95% confidence intervals did not overlap zero; these are shown in italics. “n.a.” denotes variables not included in a given model, while “+” indicates that the variable was included.


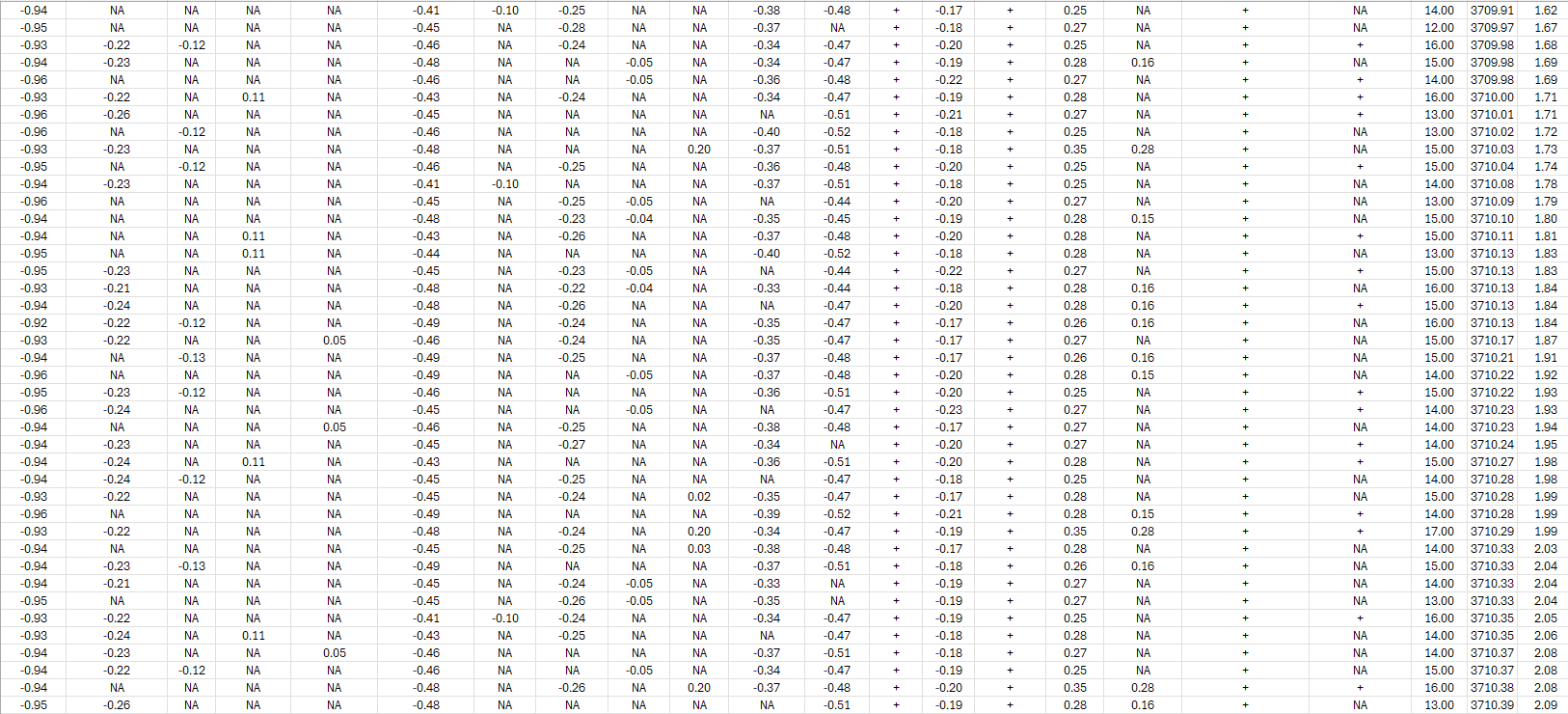


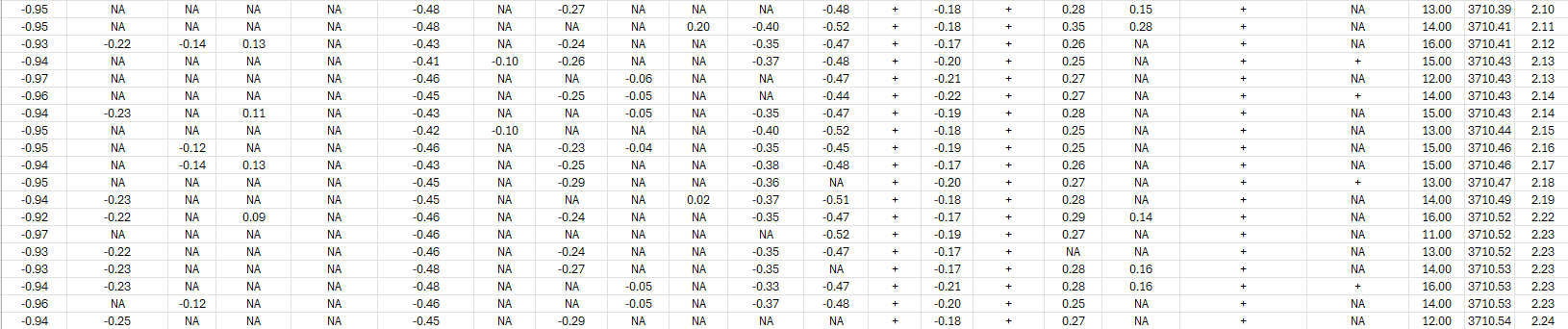

Supplement: Supplementary file 1 — Data S1: ece373215‐sup‐0003‐Supinfo.docx. [file ECE3-16-e73215-s001.docx]
